# Exploring the Potential of Synthetic Cannabinoids: Modulation of Biological Activity of Normal and Cancerous Human Colon Epithelial Cells

**DOI:** 10.3390/cells13191616

**Published:** 2024-09-26

**Authors:** Roman Paduch, Katarzyna Szwaczko, Kamil Dziuba, Adrian Wiater

**Affiliations:** 1 Department of Virology and Immunology, Institute of Biological Sciences, Faculty of Biology and Biotechnology, Maria Curie-Skłodowska University, Akademicka 19, 20-033 Lublin, Poland; roman.paduch@mail.umcs.pl; 2 Department of General and Pediatric Ophthalmology, Medical University, Chmielna 1, 20-079 Lublin, Poland; 3 Department of Organic Chemistry and Crystallochemistry, Institute of Chemical Sciences, Faculty of Chemistry, Marie Curie-Skłodowska University, Gliniana 33, 20-614 Lublin, Poland; katarzyna.szwaczko@mail.umcs.pl; 4 Department of Industrial and Environmental Microbiology, Institute of Biological Sciences, Faculty of Biology and Biotechnology, Maria Curie-Skłodowska University, Akademicka 19, 20-033 Lublin, Poland

**Keywords:** cannabinoids, colon cancer, colon normal epithelial cells, cytotoxicity, cell cycle

## Abstract

Colorectal cancer (CRC) is a global problem. Oncology currently practices conventional methods of treating this carcinoma, including surgery, chemotherapy, and radiotherapy. Unfortunately, their efficacy is low; hence, the exploration of new therapies is critical. Recently, many efforts have focused on developing safe and effective anticancer compounds. Some of them include cannabinoids. In the present study, we obtained cannabinoids, such as cannabidiol (CBD), abnormal cannabigerol (abn-CBG), cannabichromene (CBC), and cannabicitran (CBT), by chemical synthesis and performed the biological evaluation of their activity on colon cancer cells. In this study, we analyzed the effects of selected cannabinoids on the lifespan and metabolic activity of normal colonic epithelial cells and cancer colon cells. This study demonstrated that cannabinoids can induce apoptosis in cancer cells by modulating mitochondrial dehydrogenase activity and cellular membrane integrity. The tested cannabinoids also influenced cell cycle progression. We also investigated the antioxidant activity of cannabinoids and established a relationship between the type of cannabinoid and nitric oxide (NO) production in normal and cancerous colon cells. To conclude, it seems that, due to their interesting properties, the cannabinoids studied may constitute an interesting target for further research aimed at their use in alternative or combined therapies for human colon cancer.

## 1. Introduction

Colorectal cancer (CRC) is gradually becoming one of the most important medical problems in oncology. CRC is a rather diverse disease both in terms of its clinical manifestation and biological background. It is indicated that there are various symptoms, not always characteristic of CRC, as well as diverse molecular characteristics. This may lead to the detection of the tumor at various stages of advancement, which is associated with a diverse prognosis [1,2]. According to current guidelines, adenomatous polyps are the main cause of CRC development. When these changes reach a high degree of dysplasia, the risk of transformation into malignant tumors increases significantly. Therefore, in addition to prevention, understanding the basics of CRC development is a very important issue in choosing an appropriate effective treatment method. Currently, the standard in CRC therapy includes surgical resection, chemotherapy, or radiotherapy. Their effectiveness has its limitations and depends, among other factors, on the stage of development, type of tumor, and the patient’s overall response to treatment [3,4]. The limitations of chemotherapy include damage to non-cancerous rapidly dividing cells, such as the cells of the oral mucosa, bone marrow, or hair follicles. Moreover, one of the side effects of chemotherapy is neurotoxicity. In turn, radiotherapy causes both early and late side effects. The most common damages after radiotherapy include skin inflammation, damage to the skin’s secretory glands, dysfunction of the salivary glands, necrosis of the bones, lungs, heart, and genitals, and degenerations in the central nervous system. In addition, irradiation can result in the induction of secondary tumors, mainly leukemias. Therefore, the search for new and effective factors with anticancer activity is currently necessary. In addition to the already known terpenes or flavonoids, cannabinoids are one of such factors. Currently, their role is limited and focuses mainly on analgesic effects, especially related to chemical therapy. However, this does not change the attitude of scientists to studying these substances as potential factors with effective anticancer activity, mainly cytotoxic effects [5,6].

Cannabinoids are a group of terpenophenolic compounds naturally found in *Cannabis* spp. (*Cannabis sativa*), a plant with numerous valuable therapeutic properties but controversial psychotropic effects, on the other hand [7,8,9,10,11,12,13]. Biologically, the compounds are composed of geranyl diphosphate and olivetolic acid and can be extracted from plant material. Chemically, cannabinoids are easily produced by reacting olivetol (or other resorcinols) with terpene derivatives [14,15,16,17]. Currently, the anti-cancer activity of cannabinoids in experimental studies focuses mainly on induction of apoptosis and changing the population of cells in different cell cycle phases as well as inhibiting particular points of tumor cell division. Moreover, cannabinoids are known to reduce angiogenesis, mainly through disturbances in the vascular endothelial growth factor (VEGF) pathway and the inhibition of various components of this molecular trail. Consequently, the reduction in angiogenesis significantly affects the cannabinoid-related inhibition of cancer cell metastasis. This, in turn, is also associated with the modulation of the level and activity of proteases and their inhibitors by cannabinoids. Limited proteolytic activity also significantly limits the distant dissemination of cancer cells [18].

Cannabinoids have so far been used to alleviate the debilitating side effects of anti-cancer drugs, such as anorexia, pain, and vomiting. Only a few of the many natural cannabinoids known to date have undergone intensive investigations [19]. For example, cannabidiol has been shown to inhibit the progression of many types of cancer, including glioblastoma multiforme and lung, breast, prostate, and colon cancers [20]. Cannabigerol, on the other hand, promotes apoptosis, stimulates the production of reactive oxygen species, and reduces the growth of cancer cells. Studies in various experimental models of cancer have shown that cannabinoids affect the development of tumors via the endocannabinoid system (ECS). The system consists of various mediators, receptors, or transport systems and enzymes. Many studies indicate the leading role of cannabinoid receptors (CRs), especially CB1 and CB2 in the ECS. In many cancers, including colon cancer, there is abnormal functionality of this system, which may suggest its significant role in cancer development. However, special attention should also be paid to studies on the anticancer effects of cannabinoids. This indicates that the ECS itself may be a therapeutic target, and cannabinoids used as therapeutic agents may also have a strong anticancer effect [6,21,22].

Therefore, we chose cannabinoids that can be easily prepared by chemical synthesis in mild conditions and with relatively good yields using readily available preparative olivetol as a substrate, such as cannabidiol, abnormal cannabigerol, cannabichromene, and cannabicitran, to test their therapeutic potential in colon cancer. We used human HT29 colon cancer cell lines and normal cells derived from colon epithelium (CCD 841 CoTr) to determine the biological activity of synthetic cannabinoids. We also investigated their ability to reduce the free oxygen radical DPPH and the effect of cannabinoids on nitric oxide production in normal and cancerous colon cells. Our findings indicate that abn-CBG has the best cytotoxic effect. This is an important finding that has not yet been reported in the scientific literature.

## 2. Materials and Methods

### 2.1. Cannabinoid Synthesis

*General.* Commercially available chemicals were purchased from Sigma-Aldrich and used without further purification. The NMR spectra were collected with a Bruker AV500 (^1^H 500 MHz, ^13^C NMR 126 MHz) spectrometer (Bruker, Karlsruhe, Germany). All spectra were obtained in CDCl_3_ solutions, and chemical shifts (δ) were represented in ppm with internal reference to TMS. Coupling constants (*J*) were specified in Hz. The signal patterns were abbreviated as follows: s, singlet; d, doublet; t, triplet; q, quartet; m, multiplet; and b, wide. ATR-FTIR spectra were acquired using a Bruker FTIR spectrophotometer TENSOR 27 in the frequency range from 4000 to 600 cm^−1^, with a resolution of 4 cm^−1^ (Bruker Optik, Ettlingen, Germany). Melting points were determined using the Buchi 510 instrument. Thin-layer chromatography (TLC) was carried out on silica gel (Kieselgel 60, F254 on aluminum sheets, Merck, Darmstadt, Germany) under UV light (254 nm). All column chromatographic separations and purifications were performed using Merck silica gel 60 (230–400 mesh).

#### 2.1.1. Procedure for Synthesis of Olivetol

Dimethyl malonate (100.8 g, 0.62 mol), sodium methanolate (36.3 g, 0.67 mol), and 300 mL of anhydrous methanol were placed in a round-bottomed flask equipped with a mechanical stirrer and a reflux condenser. Ketone (3-nonen-2-one) was dropped into the flask, and then the mixture was heated for 4 h at 60 °C. Then, the methanol was distilled off, and the residue was dissolved in 350 mL of water and extracted with 3 × 100 mL of CHCI_3_. The aqueous layer was acidified to pH 3–4 with concentrated HCl and extracted again with 3 × 100 mL of CHCI_3_. The organic layer was dried with MgSO_4_ and, after filtering off the drying agent and distilling off CHCl_3_, the mixture was crystallized from CHCl_3_/hexane to yield 110 g of precipitate. The 110 g precipitate (0.47 mol) was placed in a round-bottom flask, and 250 mL of DMF was added. The flask was cooled with an ice–water mixture, and bromine (71.3 g, 0.44 mol), dissolved in 150 mL of DMF, was dropped into the flask. The mixture was slowly heated to 80 °C (this temperature was maintained for 3 h), and then the temperature was raised to 160 °C and maintained for another 12 h. After this time, the reaction was cooled to room temperature, and 200 mL of water was added and extracted with ether (3 × 150 mL). The organic layer was washed successively: with water, a 10% NaHSO_3_ solution, a 10% acetic acid solution, and again with water. After drying the organic fraction, the ether was evaporated, and the residue in the flask was vacuum-distilled to yield 48.0 g of pure olivetol, with a final yield of 65%. M.p. = 45–47 °C. ^1^H NMR (500 MHz, CDCl_3_): δ 6.29 (d, *J* = 2.1 Hz, 2H), 6.22 (t, *J* = 2.2 Hz, 1H), 5.96 (s, 2H), 2.43 (t, *J* = 7.9 Hz, 2H), 1.58–1.46 (m, 2H), 1.36–1.21 (m, 4H), 0.88 (t, *J* = 6.9 Hz, 3H) ^13^C NMR (126 MHz, CDCl_3_): δ 146.44, 108.27, 100.34, 35.81, 31.52, 30.73, 22.51, 14.03.

#### 2.1.2. Procedure for Synthesis of Cannabidiol

A solution of olivetol (0.5 g, 2.75 mmol), BiCl_3_ (87 mg, 0.27 mmol), and (1*S*,4*R*)-1-methyl-4-(prop-1-en-2-yl)cycloex-2-enol (0.5 g, 3.3 mmol) in 30 mL of CH_2_Cl_2_ was stirred at rt. for 1.5 h. The reaction was washed with a 10% NaHCO_3_ aqueous solution (3 × 50 mL), dried over MgSO_4_, filtered, and evaporated. The mixture was purified by column chromatography to give white solid CBD with a yield of 35% (306 mg). M.p. = 66–68 °C. ^1^H NMR (500 MHz, CDCl_3_): δ 6.32 (s, 1H), 6.18 (s, 1H), 6.04 (s, 1H), 5.62–5.58 (m, 1H), 4.90 (s, 1H), 4.68 (t, *J* = 1.8 Hz, 1H), 4.58 (d, *J* = 2.1 Hz, 1H), 3.92–3.86 (m, 1H), 2.48–2.44 (m, 2H), 2.47–2.39 (m, 1H), 2.31–2.21 (m, 1H), 2.12 (ddt, *J* = 17.8, 5.0, 2.4 Hz, 1H), 1.90–1.75 (m, 2H), 1.82 (dt, *J* = 2.4, 1.2 Hz, 3H), 1.69 (t, *J* = 1.1 Hz, 3H), 1.58 (ddd, *J* = 15.2, 8.6, 6.8 Hz, 2H), 1.32 (ddddt, *J* = 13.8, 8.6, 6.6, 5.3, 3.2 Hz, 4H), 0.91 (t, *J* = 7.0 Hz, 3H). ^13^C NMR (126 MHz, CDCl_3_): δ 156.01, 153.90, 149.28, 143.04, 140.08, 124.15, 113.79, 110.90, 109.73, 108.00, 46.21, 37.16, 35.51, 31.53, 30.68, 30.41, 28.40, 23.71, 22.57, 20.43, 14.08. IR (ATR): 3519, 3407, 3074, 2963, 2923, 2855, 1623, 1581, 1513, 1442, 1374, 1214 cm^−1^.

#### 2.1.3. Procedure for Synthesis of Abnormal Cannabigerol

A solution of olivetol (1.0 g, 5.5 mmol), *p-*TSA (95 mg, 0.55 mmol), geraniol (1.45 g, 9.4 mmol), and 100 mg of silica gel in 30 mL of CH_2_Cl_2_ was stirred at rt. for 1.5 h under an argon atmosphere. The reaction was washed with a 10% NaHCO_3_ aqueous solution (3 × 50 mL), dried over MgSO_4_, filtered, and evaporated. The mixture was purified by column chromatography to give yellow solid abn-CBG in the first fraction, with a yield of 45% (383 mg). M.p. = 61–63 °C. ^1^H NMR (500 MHz, CDCl_3_): δ 6.30 (d, *J* = 2.6 Hz, 1H), 6.26 (d, *J* = 2.5 Hz, 1H), 5.45 (s, 1H), 5.36 (s, 1H), 5.17 (tdd, *J* = 5.4, 2.7, 1.3 Hz, 1H), 5.09 (ddp, *J* = 7.0, 5.9, 1.5 Hz, 1H), 3.36–3.30 (m, 2H), 2.55 (ddd, *J* = 10.9, 5.8, 2.9 Hz, 2H), 2.18–2.03 (m, 4H), 1.83 (s, 3H), 1.71 (d, *J* = 1.4 Hz, 3H), 1.62 (d, *J* = 1.4 Hz, 3H), 1.55 (tddd, *J* = 9.6, 7.7, 4.9, 2.3 Hz, 2H), 1.36 (ddt, *J* = 7.3, 5.3, 3.0 Hz, 4H), 0.96–0.89 (m, 3H). ^13^C NMR (126 MHz, CDCl_3_): δ 155.70, 154.30, 143.33, 137.73, 131.96, 123.88, 122.74, 117.56, 108.93, 101.25, 39.68, 33.78, 31.91, 30.88, 26.46, 24.86, 22.60, 17.72, 16.23, 14.08. IR (ATR): 3345, 2954, 2922, 2855, 1619, 1587, 1458, 1348, 1285, 1126, 994 cm^−1^.

#### 2.1.4. Procedure for Synthesis of Cannabichromene

A 2-neck round bottom flask (50 mL) equipped with a distilling trap and a reflux condenser was loaded with olivetol (1.8 g, 9 mmol, 1.00 equiv.), citral (1.7 mL, 10 mmol, 1.1 equiv.), and toluene (20 mL), and next polymer-bound tris(2-aminoethyl)amine (0.32 g, 1.35 mmol, 15 mol%) was added. The resulting solution was stirred at reflux while being monitored by HPLC analysis until it showed no further signs of consuming the remaining olivetol. The reaction mixture was then cooled to room temperature. The reaction mixture was filtered through a pad for removing the amine-catalyst, and the solvent was removed under vacuum. The yellow crude oil was purified using column chromatography to obtain 1.75 g (62%) of yellow oil of CBC. ^1^H NMR (500 MHz, CDCl_3_): δ 6.65 (dd, *J* = 10.0, 0.8 Hz, 1H), 6.29–6.27 (m, 1H), 6.15 (d, *J* = 1.4 Hz, 1H), 5.52 (d, *J* = 10.0 Hz, 1H), 5.12 (dddd, *J* = 7.2, 5.8, 2.9, 1.4 Hz, 1H), 4.97 (s, 1H), 2.49–2.44 (m, 2H), 2.17–2.09 (m, 2H), 1.80–1.62 (m, 2H), 1.69 (d, *J* = 1.3 Hz, 3H), 1.60 (d, *J* = 1.3 Hz, 3H), 1.59–1.55 (m, 2H), 1.41 (s, 3H), 1.35–1.29 (m, 4H), 0.93–0.89 (m, 3H). ^13^C NMR (126 MHz, CDCl_3_): δ 154.03, 151.04, 144.76, 131.65, 127.22, 124.21, 116.84, 109.10, 107.71, 107.00, 78.19, 41.05, 35.92, 31.49, 30.66, 26.26, 25.70, 22.73, 22.56, 17.64, 14.04. IR (ATR): 3395, 2958, 2926, 2856, 1621, 1578, 1428, 1376, 1083, 1053 1033 cm^−1^.

#### 2.1.5. Procedure for Synthesis of Cannabicitran

A 2-neck round bottom flask (100 mL), equipped with a distilling trap and a reflux condenser, was loaded with olivetol (3.6 g, 18 mmol, 1.00 equiv.), citral (3.4 mL, 20 mmol, 1.1 equiv.), and toluene (125 mL), and next polymer-bound tris(2-aminoethyl)amine (0.64 g, 2.7 mmol, 15 mol%) was added. The resulting solution was stirred at reflux while being monitored by HPLC analysis until it showed no further signs of consuming the remaining olivetol. The reaction mixture was then cooled to room temperature. The reaction mixture was filtered through a pad to remove the amine-catalyst, and the solvent was removed under vacuum. Then, the reaction mixture was distilled under vacuum at 0.1 mbar, but the crude oil bubbled for some time. The yellow oil began to distill at about 115 °C (0.1 mbar) to obtain 2.32 g (41%) of brown oil of CBT. ^1^H NMR (500 MHz, CDCl_3_): δ 6.35 (d, *J* = 0.8 Hz, 1H), 6.30 (d, *J* = 1.3 Hz, 1H), 2.87 (dt, *J* = 4.5, 2.0 Hz, 1H), 2.55–2.50 (m, 2H), 2.24 (ddd, *J* = 13.2, 4.7, 3.1 Hz, 1H), 2.04 (ddd, *J* = 11.5, 5.3, 2.8 Hz, 1H), 1.85 (dd, *J* = 13.1, 1.7 Hz, 1H), 1.78 (dddd, *J* = 14.9, 6.2, 3.2, 1.4 Hz, 1H), 1.63–1.55 (m, 2H), 1.54 (s, 3H), 1.44 (ddd, *J* = 13.1, 8.1, 6.6 Hz, 1H), 1.40 (s, 3H), 1.36–1.21 (m, 5H), 1.03 (s, 3H), 0.91–0.87 (m, 3H), 0.63 (tdd, *J* = 13.5, 11.5, 6.1 Hz, 1H). ^13^C NMR (126 MHz, Chloroform-d) δ 156.87, 156.56, 142.57, 124.26, 114.09, 109.80, 108.92, 83.61, 74.51, 46.79, 37.36, 36.16, 35.37, 31.43, 31.02, 29.76, 29.10, 28.12, 23.76, 22.56, 22.16, 14.06. IR (ATR): 2970, 2954, 2927, 2872, 2856, 1619, 1585, 1429, 1365, 1162, 1128, 1086 cm^−1^.

### 2.2. Biological Effects of Cannabinoids

#### 2.2.1. Cell Cultures

The HT29 cell line (ATCC no. HTB-38) (human colon adenocarcinoma) was used in the present study. Cells were cultivated in RPMI 1640 culture medium with 10% fetal calf serum (FCS) (GibcoTM, Paisley, UK), supplemented with antibiotics (100 U/mL penicillin and 100 μg/mL streptomycin) (Sigma, St. Louis, MO, USA). The cell culture was performed in standard conditions, i.e., at 37 °C in a humidified atmosphere with 5% CO_2_. The CCD 841 CoTr cell line (ATCC no. CRL-1807) (human normal colon epithelium) was cultivated in RPMI 1640 +DMEM (1:1) medium (Sigma, St. Louis, MO, USA) with 10% FCS and the addition of antibiotics. The culture was carried out at 34 °C in a 5% CO_2_/95% air atmosphere.

#### 2.2.2. MTT Assay

After seeding (1 × 10^5^ cells/mL) in a 96-well plate, the cells were incubated for 24 h. Thereafter, the tested compounds (100 μL) at a concentration range of 0–200 μg/mL in the culture medium were added to the wells (100 μL). The incubation lasted for 24 h. After that time, an MTT solution (5 mg/mL, 25 μL/well) was added to each well. The incubation was continued for another 3 h. After the incubation, 10% sodium dodecyl sulfate (SDS) in 0.01 M HCl (100 μL) was added in order to dissolve the purple formazan crystals. Solubilization was carried out overnight. Finally, spectrophotometric measurement was performed at 570 nm using an EL800 Universal Microplate Reader (BioTek Instruments, Winooski, VT, USA).

#### 2.2.3. Neutral Red (NR) Uptake Assay

After seeding (1 × 10^5^ cells/mL) in a 96-well plate, the cells were incubated for 24 h. Thereafter, the tested compounds (100 μL) at a concentration range of 0–200 μg/mL in the culture medium were added to the wells (100 μL). The incubation lasted for 24 h. After that time, the culture medium was removed and a 0.4% Neutral Red solution in the culture medium was added to each well. The incubation was continued for another 3 h. After the incubation, the medium with the NR dye was discarded, and 4% paraformaldehyde in 1% CaCl_2_ was added for cell fixation (200 μL) and incubated for 3 min. Intracellular dye deposits were solubilized using 1% acetic acetate in a 50% ethanol solution (100 μL). The plates were then gently shaken (20 min.) at room temperature to dissolve and release the dye from the cells. Finally, spectrophotometric measurement was performed at 540 nm using an EL800 Universal Microplate Reader (BioTek Instruments, Winooski, VT, USA).

#### 2.2.4. Cytometric Analysis of the Cell Cycle

In the flow cytometric study, we assessed the impact of the cannabinoids at a concentration of 25 μg/mL on the distribution of cell cycle phases in the normal and cancer cells after 24 h incubation. Both adhered, and non-adsorbed (floating) cells were used to perform the analyses. They were collected after 24 h of incubation with the tested cannabinoids and centrifuged (3000 rpm/5 min.). Next, the cell pellet was rinsed and suspended in PBS w/o Ca^2+^ and Mg^2+^ ions and centrifuged again (3000 rpm/5 min.). The cell pellet was suspended and fixed in 70% ethanol (1 mL). Untreated cells served as a control. The cells were stored at −20 °C for 1 week. After that time, in accordance with the manufacturer protocol, the cells were subjected to the PI staining procedure (PI/RNase Staining Buffer, BD PharmingenTM, BD Biosciences, San Jose, CA, USA). Briefly, 1 mL of PBS w/o Ca^2+^ and Mg^2+^ ions was added to 1 mL of ethanol and incubated with gentle stirring for 5 min. at room temperature. Next, the cells were centrifuged (3000 rpm/5 min.) and suspended in PBS w/o Ca^2+^ and Mg^2+^ ions with gentle stirring for 5 min. at room temperature. The cells were centrifuged once again (3000 rpm/5 min.) and the cell pellet was suspended in 500 μL of PI/RNase Staining Buffer. The incubation was carried out for 15 min. at room temperature. Finally, the intensity of the PI fluorescence was measured using FACS Calibur (BD Pharmingen^TM^). The obtained data were analyzed using Cell Quest Pro Version 6.0. for the Macintosh operating system (BD Pharmingen^TM^). The percentage of cells in the appropriate phases of the cycle was calculated based on all analyzed gated cells. A total of 10,000 events were measured per sample.

#### 2.2.5. Flow Cytometry–Quantitative Analysis of Cell Death Types

In a further flow cytometric study measuring normal and tumor cell death types, the cells were exposed to the cannabinoids at a concentration of 25 μg/mL for 24 h.

The annexin V-fluorescein isothiocyanate (FITC)/propidium iodide (PI) apoptosis kit (BD Biosciences, BD Pharmingen™, San Jose, CA, USA) was used for the quantitative analysis of cannabinoid-induced cell death.

For the analyses, both adhered (after detaching the cells with 10 mM EDTA in PBS w/o Ca^2+^ and Mg^2+^ ions solution) and floating cells were collected after 24 h of incubation with the tested cannabinoids. After collection, the cells were gently centrifuged (1000 rpm/5 min.). Next, the supernatant was discarded, and the cell pellet was suspended in warm PBS w/o Ca^2+^ and Mg^2+^ ions and incubated for 5 min. at room temperature. The centrifugation procedure was repeated again and, following the manufacturer’s procedure, the cells were suspended in 100 μL of 1X Annexin V Binding Buffer with 5 μL of FITC Annexin V (5 mM) and 5 μL of propidium iodide (PI) (5 mM). The preparation was gently vortexed and incubated at room temperature for 15 min. in the dark. After that time, the cells were subjected to cytometric analysis. Before dosing the cells into the cytometer, 400 μL of 1X Binding Buffer was added. The cells were analyzed using a flow cytometer (BD FACSCalibur) with CellQuest Pro Version 6.0 software within 1 h after the staining was completed.

#### 2.2.6. Nitric Oxide (NO) Measurement

In this spectrophotometric method, the stable end products (nitrate and nitrite) were analyzed in the normal and tumor culture supernatants after 24 h of incubation of the cells with the cannabinoids in the concentration range of 0–200 μg/mL. The Griess reaction was applied. This method involves a 2-step reaction, whose product is a red-pink azo dye. The amount of this dye is directly proportional to the amount of NO. The cells were cultured for 24 h in 24-well plates (1 mL/well, density 1 × 10^5^ cells/mL); next, after withdrawing the culture fluid, the tested cannabinoids were added to the wells at a volume of 1 mL/well and the incubation was carried out for another 24 h. After the incubation, the culture media were collected and frozen at −80 °C for no longer than 1 month. For calibration and quantitative analysis, the standard curve was prepared using 0.5–25 μM sodium nitrite (NaNO_2_). Thawed and centrifuged (5000 rpm/5 min.) supernatant samples and the standard were poured into a 96-well flat-bottomed plate at 100 µL/well. Each sample was analyzed in triplicate. Subsequently, 100 µL/well of Griess reagent (1% sulfanilamide/0.1% *N*-(1-naphthyl)ethylenediamine dihydrochloride) in 3% H_3_PO_4_ was added to all wells and incubated at room temperature for 10 min. Finally, the absorbance of the samples was spectrophotometrically read at 570 nm using an EL800 Universal Microplate Reader (BioTek Instruments, Winooski, VT, USA).

#### 2.2.7. DPPH Free Radical Scavenging Test

The DPPH biochemical test is used to determine the antioxidant potential of tested compounds. This method is based on the ability of compounds to reduce the stable dark purple 2,2-diphenyl-1-picrylhydrazyl radical (DPPH) to yellow diphenyl-picrylhydrazine. The following concentrations of the tested cannabinoids were prepared: 25, 100, and 200 μg/mL in pure ethanol. Next, the DPPH solution (0.2 mg/mL in ethanol) was prepared. As a quantitative standard, Trolox in increasing concentrations (1–50 μg/mL) was prepared in ethanol. The procedure was carried out as follows: 100 µL of the specified concentrations of the tested cannabinoids or standard points were added to the wells of a 96-well plate in eight replicates. Next, 100 μL of a DPPH solution was added to 100 μL of each sample or standard in a 96-well plate. Subsequently, 20 min. incubation at room temperature was carried out, and spectrophotometric reading was performed at 515 nm using an EL800 Universal Microplate Reader (BioTek Instruments, Winooski, VT, USA). Decreasing absorbance meant increasing antioxidant activity of the preparations. The final value of the free radical scavenging activity of the cannabinoids was calculated based on the standard curve and the following formula:

DPPH scavenging effect (%) = [(X_control_ − X_compound_/X_control_) × 100]

X_control_ is the absorbance of the control, and X_compound_ is the absorbance in the presence of cannabinoids

#### 2.2.8. Ferric-Reducing Antioxidant Power Assay (FRAP)

The antioxidant activity in the FRAP method determines the ability of the analyzed compounds to reduce iron ions. This allowed for the direct determination of the reducing capacity of the tested cannabinoids. The principle of the method was based on measuring the reduction in the tPtZ compound (iron-2,4,6-tripyridyl-S-thiazine complex) under the influence of the cannabinoids. The following concentrations of the cannabinoids were prepared: 25, 100, and 200 μg/mL in Milli-Q water. As a quantitative standard, ascorbic acid in increasing concentrations (0–150 μg/mL) was prepared in Milli-Q water. The procedure was carried out as follows: each compound, at a concentration of 1 mL, was mixed with 1 mL of 0.2 M sodium phosphate buffer (pH 6.6) and 1 mL of 1% potassium ferricyanide. The mixture was vortexed and incubated for 30 min. at 37 °C. After the incubation, 1 mL of 10% trichloroacetic acid (*w*/*v*) was added to each sample, and the preparations were centrifuged at 1000 *g* for 5 min. Next, an equal volume of Milli-Q water and the upper layer of the samples (1 mL + 1 mL) were mixed with 100 μL of 0.1% ferric chloride and subjected to spectrophotometric reading at 700 nm using an EL800 Universal Microplate Reader (BioTek Instruments, Winooski, VT, USA). The higher the absorbance, the higher the iron ion-reducing activity of the tested cannabinoids is.

#### 2.2.9. Statistical Analysis

All experiments were performed in triplicate, and the results are presented as means ± Standard Deviation. One-way ANOVA with Dunnett’s post hoc test was used for data analysis. Differences with *p *≤ 0.05 were considered significant.

## 3. Results

### 3.1. Synthetic Strategies Adopted in the Preparation of Cannabinoids

**Scheme 1:**
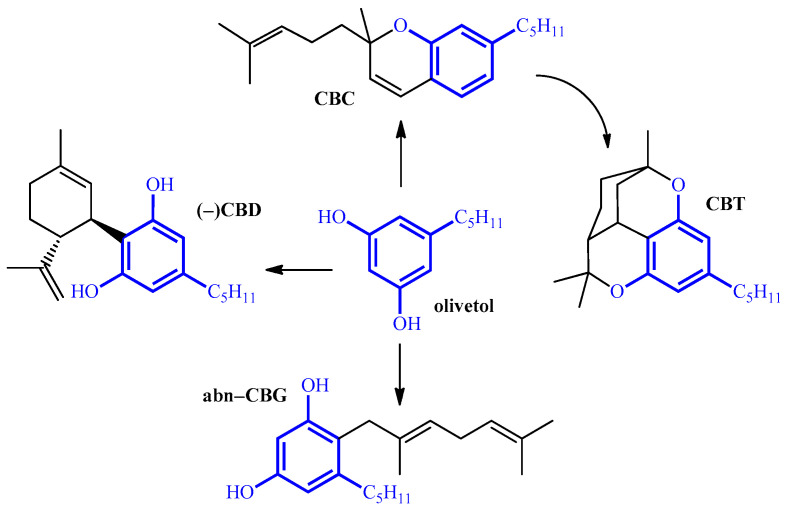
Synthesis route used for the preparation of cannabinoids.

The CBD and CBG cannabinoids were synthesized according to procedures known in the literature. Olivetol is a key building block in the chemical synthesis of phytocannabinoids and their intermediate analogs [23,24,25]. The efforts to chemically synthesize the above-mentioned cannabinoids primarily involve the regioselective terpenylation of olivetol (Scheme 1).

**Scheme 2:**
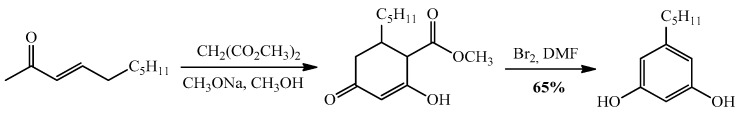
Two-step synthesis of olivetol.

To synthesize olivetol, we used a two-reaction sequence (Scheme 2) [26], starting with the corresponding *α,β*-unsaturated ketone, which was subjected to reaction with diethyl malonate in base conditions to afford a cyclic Michael adduct. In the second step, the cyclic intermediate compound was heated with a solution of bromine in dimethylformamide (DMF) to produce the target olivetol, with a final yield after vacuum distillation of up to 65%.

**Scheme 3:**
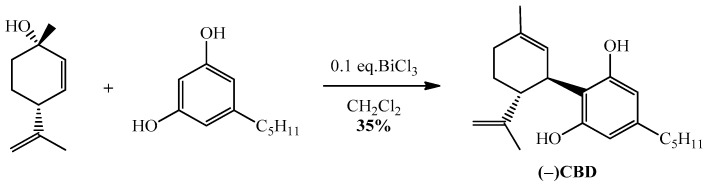
Synthesis of CBD from olivetol and (1*S*,4*R*)-1-methyl-4-(prop-1-en-2-yl)cycloex-2-enol.

CBD, CBG, and abn-CBG (abnormal CBG) can be prepared using several synthetic methods [27,28,29,30]. The most widely exploited and readily available synthetic procedure involves a one-step Friedel–Craft allylation of olivetol with a suitable chiral terpene in the presence of Brönsted or Lewis acid catalysts [31]. For our synthesis, (-)CBD was achieved by the stereoselective reaction of olivetol with (1*S*,4*R*)-1-methyl-4-(prop-1-en-2-yl)cycloex-2-enol employing 0.1 eq. BiCl_3_ (Scheme 3). In methylene chloride, after 90 min., the target cannabinoid was obtained, with a final yield of 35%.

**Scheme 4:**
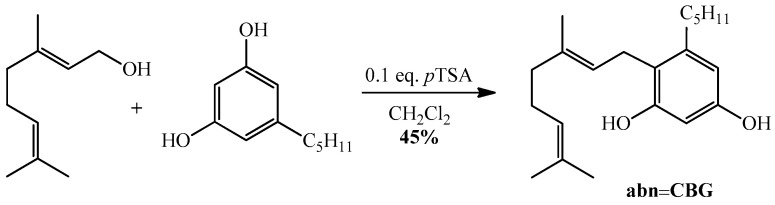
Synthesis of abn-CBG from olivetol and geraniol.

The regioisomer of natural CBG, abnormal CBG, was prepared via the Lewis acid-catalyzed reaction of olivetol with geranyl. The reaction was performed in methylene chloride at room temperature in the presence of silica gel for 1.5 h. The use of p-toluenesulfonic acid (*p*TSA) in an amount of 0.1 eq as a catalyst was found to produce the target compound in satisfactory yields of up to 45% (Scheme 4). Pure abn-CBG was isolated from the reaction mixture by column chromatography.

**Scheme 5:**
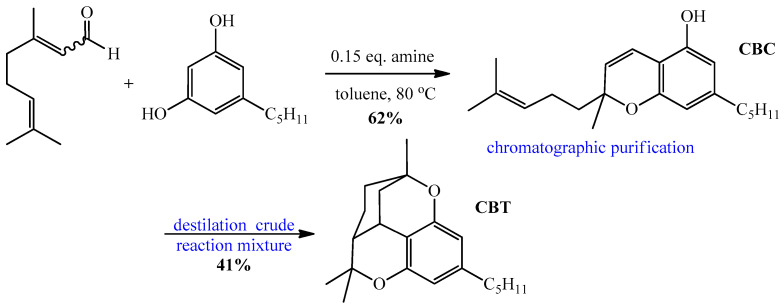
Synthesis of CBC and CBT from olivetol and citral.

We synthesized CBC using the known method [32], but we chose tris(2-aminoethyl)amine supported on the polymer as a recoverable catalyst to increase the yield and simplify the purification process. We were also able to prepare CBT in a similar way, but instead of using chromatography to separate the product, as described above, we distilled the reaction mixture under vacuum; in this way, CBC isomerizes thermally to pure CBT with a high yield (Scheme 5).

### 3.2. Metabolic Activity of Tumor and Normal Colonic Cells after Incubation with Tested Cannabinoids: MTT Test

**Figure 1:**
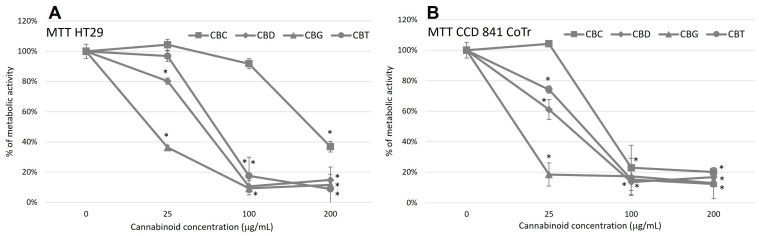
MTT analysis of the metabolic activity of HT29 cells (**A**) and CCD 841 CoTr cells (**B**) after 24 h of incubation with the tested cannabinoids. * A *p*-value of ≤0.05 is considered significant.

The tested cannabinoids showed activity in reducing the metabolism of HT29 cancer cells depending on their concentration. The abn-CBG preparation had the strongest effect, as it reduced the metabolic activity of cells by 50% at a concentration of just 19.61 μg/mL. The weakest effect was recorded for the CBC sample, for which the IC50 was 175.96 μg/mL (Figure 1A).

The tested cannabinoids also influenced the metabolism of normal human colon epithelial cells (CCD 841 CoTr). At a concentration of 25 μg/mL, the differences in the activity of the individual preparations were most visible. The weakest effect was observed for the CBC preparation, which at the lowest concentration even stimulated cell metabolism by over 4%, compared to the control. At higher concentrations, a sharp decrease in cell metabolism was observed. The highest IC50 value, i.e., 75.25 μg/mL, was demonstrated for the CDC preparation. The strongest activity was demonstrated by the abn-CBG preparation, which at a concentration of 25 μg/mL reduced the metabolism of normal cells by over 81%. The IC50 for the abn-CBG preparation was 15.34 μg/mL (Figure 1B and Table 1).

### 3.3. Viability of Tumor and Normal Colonic Cells after Incubation with Tested Cannabinoids. Neutral Red (NR) Uptake Assay

**Figure 2:**
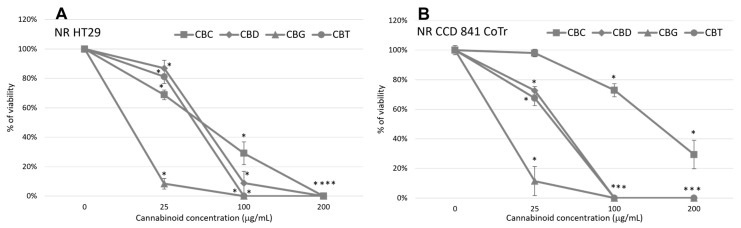
NR uptake assay analysis of the viability of HT29 cells (**A**) and CCD 841 CoTr cells (**B**) after 24 h of incubation with the tested cannabinoids. * A *p*-value of ≤0.05 is considered significant.

At increasing concentrations, the tested cannabinoids reduced the viability of colorectal cancer cells. The abn-CBG preparation had the strongest effect, with an IC50 of 13.66 μg/mL. The other cannabinoids reduced the viability of HT29 cells in a similar way, showing an IC50 in the range of 54–61 μg/mL (Figure 2A).

The normal colonic epithelium cells showed varying sensitivity to the tested cannabinoids. The CBC preparation had the weakest effect, with an IC50 of 150.86 μg/mL. The strongest activity was demonstrated by the abn-CBG preparation, whose IC50 was 14.12 μg/mL. The CBD and CBT preparations with similar activity reduced the viability of normal cells, with an IC50 of 48.25 and 44.46 μg/mL, respectively (Figure 2B and Table 2).

### 3.4. Viability and Types of Tumor and Normal Colonic Cell Death after Incubation with Tested Cannabinoids: Cytometric Analysis

**Figure 3:**
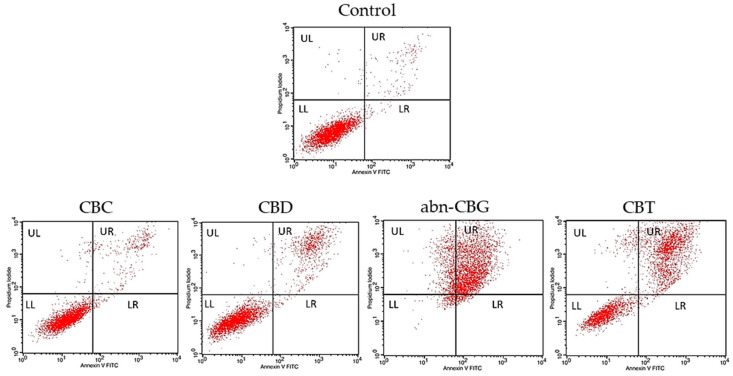
Apoptosis/necrosis level after the incubation of HT29 tumor cells with the cannabinoids at the 25 μg/mL concentration. Flow cytometry analysis after cell staining with annexin V-FITC and propidium iodide. Only representative dot plots are presented. An−/PI+ necrotic cells are located in the UL Quad, An+/PI+ late apoptotic cells in the UR Quad, An−/PI− viable cells in the LL Quad, and An+/PI− early apoptotic cells in the LR Quad.

The CBC and CBD preparations reduced the number of viable cancer cells by 7.13% and 13.62%, respectively, compared to the control. The main pathway of cell death was apoptosis, and most cells showed signs of late apoptosis after 24 h of incubation. The abn-CBG preparation was the most efficient in reducing the number of viable cells (by 90.42%, compared to the control). Mainly, cells in late apoptosis (68.83% more than in the control) and necrosis (19.03% more than in the control) were observed. The CBT cannabinoid reduced the number of viable cells by 52.25%, compared to the control. The predominant number of dying cells was in the phase of late apoptosis (49.2% more than in the control). The other dead cells were the necrotic ones (3.24% more than in the control) (Figure 3 and Table 3).

**Figure 4:**
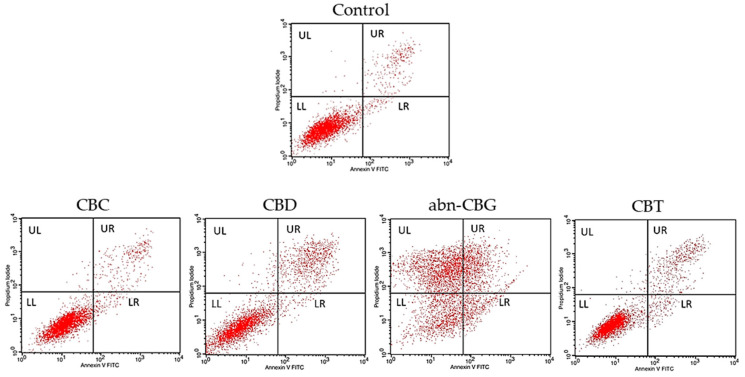
Apoptosis/necrosis level after the incubation of CCD 841 CoTr normal cells with the cannabinoids at the 25 μg/mL concentration. Flow cytometry analysis after cell staining with annexin V-FITC and propidium iodide. Only representative dot plots are presented. An−/PI+ necrotic cells are located in the UL Quad, An+/PI+ late apoptotic cells in the UR Quad, An−/PI− viable cells in the LL Quad, and An+/PI− early apoptotic cells in the LR Quad.

The CBC preparation did not significantly affect the distribution of normal cell viability, compared to the control. The CBD, abn-CBG, and CBT preparations significantly reduced the number of viable normal cells by 12.95%, 66.79%, and 5.02%, respectively, compared to the control. In the case of the CBD cannabinoid, the cells died mainly by late apoptosis (11.36% more than in the control) and necrosis (1.43% more than in the control). The abn-CBG preparation induced both phases of apoptotic cell death, with early and late apoptosis accounting for 19.52% of dying cells, compared to the control, and necrosis accounting for 47.27% of dying cells, compared to the control. The CBT preparation mainly induced apoptosis, increasing the number of cells in late apoptosis (3.12% of dying cells more than in the control) after 24 h of incubation (Figure 4 and Table 4).

### 3.5. Flow Cytometry Analysis of Tumor Cells and Normal Colonic Epithelium Cell Cycle Changes after Incubation with Tested Cannabinoids

**Figure 5:**
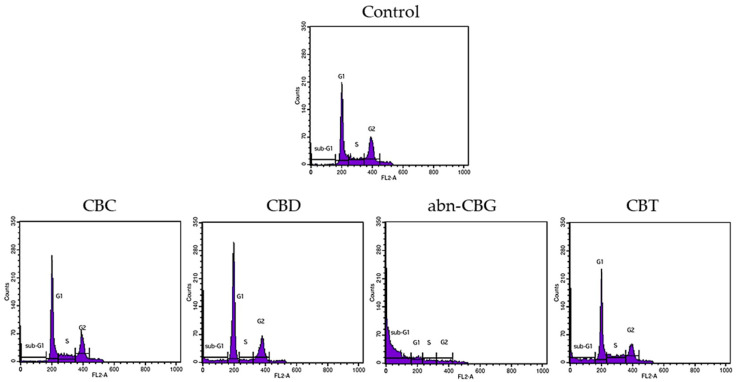
Flow cytometry analysis of changes in the cell cycle phases in HT29 human colon tumor cells after 24 h of incubation with the tested cannabinoids (25 μg/mL). Only representative DNA histograms are presented.

The studied cannabinoids significantly influenced the phases of the cancer cell cycle. The CBC preparation increased the number of cells in the sub-G1 phase by 1.19% and reduced the number of cells in the G2 phase by 3.24%, compared to the control. The CBD preparation increased the number of cells in the sub-G1 phase by 7.26% and significantly limited the transition between the G1/S phases, compared to the control. The abn-CBG preparation showed mainly toxic effects, which was reflected in an 80.59% increase in the number of cells in the sub-G1 phase, compared to the control. The CBT cannabinoid also increased the number of cells in the sub-G1 phase (an increase of 17.93%, compared to the control) and limited the transition of cells from the S phase to the G2 phase (Figure 5 and Table 5).

**Figure 6:**
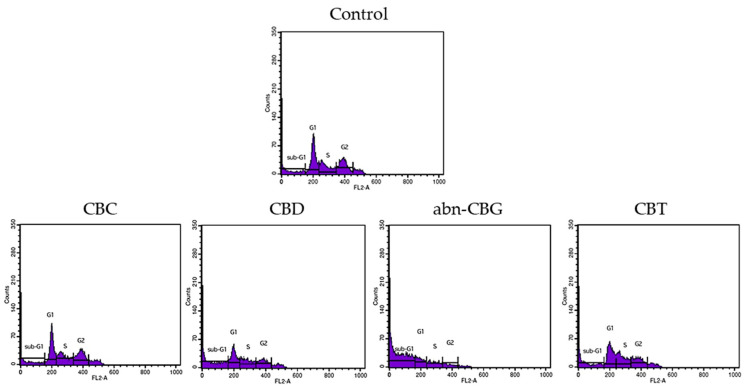
Flow cytometry analysis of changes in the cell cycle phases in CCD 841 CoTr human colon normal cell after 24 h of incubation with the tested cannabinoids (25 μg/mL). Only representative DNA histograms are presented.

The tested cannabinoids also significantly influenced changes in the distribution of normal cells in the cell cycle phases. CBC reduced the number of cells in the sub-G1 phase by 6.96%, compared to the control, which indicates an anti-apoptotic effect, and limited the transition of cells from the S to G2 phase. CBD significantly increased the number of cells in the sub-G1 phase by 21.88%, and thus significantly reduced the number of cells in the other phases, compared to the control. The abn-CBG activity was mainly related to the accumulation of cells in the sub-G1 phase (an increase of 53.97%, compared to the control), which indicates, similarly to the CBD preparation, a pro-apoptotic effect. The CBT preparation increased the number of cells in the sub-G1 phase by 6.71% and caused the accumulation of cells in the S phase, compared to the control (Figure 6 and Table 6).

### 3.6. Nitric Oxide (NOx) Secretion in the Culture of HT29 and CCD 841 CoTr Cells after Incubation with Cannabinoids

**Figure 7:**
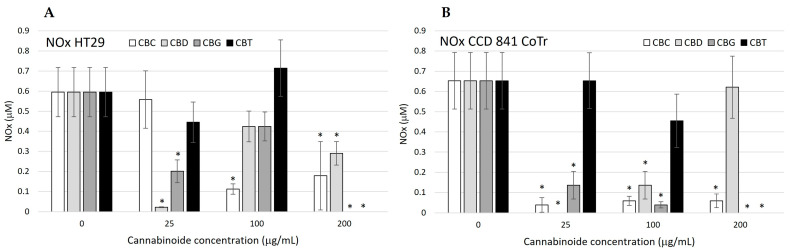
Level of nitric oxide (NOx) released in the culture of HT29 (**A**) and CCD 841 CoTr (**B**) cells, analyzed with the Griess method. Cells were incubated for 24 h with the cannabinoids (0–200 μg/mL). The columns show the mean and bars ± SD. The experiments were repeated three times. A * *p*-value of ≤0.05 is considered significant. The control NOx level is compared to the samples.

The tested cannabinoids influenced the release of nitric oxide NOx by the tested cancer (Figure 7A) and normal cells (Figure 7B). A reduction in NOx levels with increasing cannabinoid concentration was induced by the CBC preparation. The CBD preparation caused an increase in NOx levels with increasing concentration, but only in normal cells. The CBT preparation at concentrations up to 100 μg/mL had a statistically insignificant stimulating effect on the release of NOx in cancer cells, while it reduced the level of this molecule in normal cells. A similar pattern of action was observed for the abn-CBG preparation, but the differences were statistically significant.

### 3.7. DPPH Scavenging Activity at Different Concentrations of Tested Cannabinoids

The CBC cannabinoid applied at the lower concentrations (<100 μg/mL) did not express any activity in reducing the free oxygen radical DPPH. The highest reduction, increasing with the increasing cannabinoid concentration, was demonstrated for the CBT sample (a concentration of 200 μg/mL CBT, corresponding to Trolox activity at a concentration of 0.593 μg/mL). Activity was also demonstrated at the lowest CBT concentration (25 μg/mL), corresponding to the synthetic activity of vitamin E at a concentration of 0.280 μg/mL. Similar activity was found for the cannabinoid designated as CBD, but its lowest concentration induced no DPPH-reducing activity. At the highest concentration applied (200 μg/mL), the abn-CBG cannabinoid showed DPPH radical reducing activity equivalent to 0.216 μg/mL of Trolox (Table 7).

### 3.8. Ferric-Reducing Antioxidant Activity by Different Concentrations of Tested Cannabinoids

All the tested cannabinoids had the ability to reduce Fe^3+^ iron ions. The weakest effect was observed for the CBD sample, which, at the highest concentration applied (200 μg/mL), showed reducing activity, corresponding to 0.202 μg/mL of ascorbic acid. The strongest reducing properties were demonstrated by the abn-CBG preparation, which, at a concentration of 200 μg/mL, showed activity equivalent to 1.411 μg/mL of vitamin C (Table 8).

## 4. Discussion

Cannabinoids in the therapeutic context are currently used mainly as an analgesic agents. Their use in chemotherapy or for the inhibition of cancer cell invasiveness is quite insignificant. Nevertheless, there are reports of the effectiveness of cannabinoids in reducing the viability of cancer cells of various origins, including breast, lung, prostate, colon cancers, as well as glioma, neuroblastoma, and myeloma [33]. In this study, we presented the activity of four cannabinoids: CBD (cannabidiol), abn-CBG (abnormal cannabigerol), CBC (cannabichromene), and CBT (cannabicitran) on human colon cancer and normal cells cultured in vitro.

Colon cancer is one of the major malignancies detected in the field of oncology. The cells of this tumor demonstrate rapid development and acquisition of the ability to metastasize. Despite their effects, the therapeutic methods used currently do not always prove effective in combating the development of this type of tumor, hence the need to search for new methods and factors exhibiting the desired activity. Cannabinoids exhibit quite interesting properties aimed at modulating the proliferation of tumor cells, limiting the process of tumor vascularization, and thus limiting tumor spread [3,4]. In addition, cannabinoids have properties that alleviate the side effects caused by chemotherapy and can be used in potential combination therapies, increasing the effectiveness of some anticancer drugs [34].

Therefore, cannabinoids are gaining popularity as a supportive therapy in cancer treatment, often in combination with standard chemotherapeutics. Compounds of this group are therefore an interesting alternative in treatment, additionally demonstrating immunomodulatory activity. Cannabinoids demonstrate activity through their CB1 and CB2 receptors. They are differentially expressed on cancer cells, depending on the origin of the pathological tissue. In the case of colon cancer, CB1 and CB2 receptor overexpression is related to a poor disease outcome. Therefore, cannabinoid activity can be associated with cancer prognosis [6,22]. The binding of ligands to cannabinoid receptors inhibits the proliferation of cancer cells and leads to their death via apoptosis. Cannabinoids affect the P13/AKT/mTOR axis responsible for regulating cell growth and survival. Another pathway activated by cannabinoids, limiting cancer cell proliferation, is the MAPK pathway [33]. In our study, we demonstrated the influence of the tested cannabinoids on the viability and metabolic activity of normal colonic epithelial cells and cancer cells derived from the intestinal epithelium. This is in agreement with data presented by Buchtova et al. and Pisanti et al., who cited data indicating that cannabinoids, specifically CBD, have an antiproliferative effect on breast cancer cells. The effect of CBD activity was mainly mediated by the influence of this factor on CB1 and CB2 receptors. The effect observed in our study may also be based on this mechanism [34,35]. The cytotoxic activity of CBD and CBG on the colon cancer cell line SW-620 was also confirmed by Bęben et al. They, however, showed stronger activity of these cannabinoids on SW620 cells compared to that observed in our experiments. This confirms the dependence of the activity on the concentration and type of cannabinoid and the tissue on which it acts [36]. Aviello et al. also observed that cannabidiol is characterized by proapoptotic activity and inhibition of colon cancer cell proliferation [5,37]. Cancer cell apoptosis induced by cannabinoids may be associated with the enzyme caspase 3/7. In addition, free oxygen radicals (ROS) may play an important role. The mitochondrial membrane potential is then changed, triggering the release of cytochrome c [38]. In our study, we demonstrated the effect of cannabinoids on the activity of mitochondrial dehydrogenase and its importance in reducing the viability of cancer cells, but also normal colon cells. Therefore, changes in mitochondrial activity may also be connected with the toxic effects of cannabinoids. Moreover, in vitro studies on a colon cancer model have shown that the use of CBD leads to cell apoptosis mainly through changes in the expression of pro- and antiapoptotic proteins affecting the tumor mass [5,39]. However, in our study, we showed relatively low proapoptotic and cytotoxic activity of CBC and CBD. It has been reported that cannabidiol exerts low toxic effects and is described as having a good safety profile [40,41]. Therefore, it is a promising compound in pharmacology.

Studies by Iden et al. indicate that the endogenous activation of CB2 may modulate the immune response and limit tumorigenesis. This indicates that CB2 may have an antitumor role in colon cancer and may be a therapeutic target in this tumor [2]. It is also indicated that cannabinoid extracts also have a potential cytotoxic effect on colon polyps. It has been shown that cannabigerol (CBG) can inhibit colon carcinogenesis via apoptosis promotion, which is also associated with higher ROS stimulation [1,42]. This is in agreement with our results, showing an increased apoptosis of the tumor cells after the abn-CBG application, as well as changes in nitric oxide, which may also serve as a reactive radical. Studies conducted on ovarian cancer cells indicated that CBD and abn-CBG induced time- and concentration-dependent cytotoxicity. In our study, abn-CBG also showed the highest cytotoxic activity. Ben-Ami Shor also showed similar toxicity towards colonic polyps and normal adjacent epithelial cells [1]. This agrees with our results, where the sensitivity of normal and tumor cells to the tested cannabinoids was comparable. This may indicate the usefulness of this cannabinoid in potential therapies. We, however, showed its strong action on normal cells, which encourages the consideration of reducing the concentration range and determining an optimal level, with reduced toxicity towards normal cells.

Studies have shown that cannabinoids also exhibit antioxidant activity [43,44]. They chelate free oxygen radicals and transform them into less active forms. The extent of their antioxidant activity may depend on the number of phenol hydroxyl groups in the studied types of cannabinoids [43,45]. Preparations containing cannabinoids from, e.g., *Cannabis sativa* leaves, exhibit antioxidant activity, which grows with the increasing total amount of cannabinoids in the preparation [46,47]. In our study, we also found that the tested cannabinoids exhibited antioxidative activity. Similar observations were reported by Borrelli et al., who found that cannabidiol reduced reactive oxygen species (ROS) and inducible nitric oxide synthase (iNOS) in the human colon adenocarcinoma (CaCo-2) cell line [48]. Cannabidiol is generally considered an anti-colon cancer compound due to its antimetastatic activity and the ability to increase the action of antioxidant enzymes [49]. Nitric oxide (NO), which belongs to the group of reactive nitrogen species, has a strong effect on the growth and survival of cancer cells. Romano et al. showed that CBC reduced the level of nitric oxide in peritoneal macrophages activated by LPS. It has also been indicated that NO production can be modulated by CB1 receptors [50]. Similar conclusions were obtained by Carney et al., indicating the promotion of NO production by the CB1 receptor in biological systems [51]. In turn, in studies on cartilage, cannabinoids have been shown to protect chondrocytes by inhibiting NO production [52]. The differential effect of cannabinoids on nitric oxide release by murine macrophages was observed by Yekhtin et al. This effect was connected with alleviated expression of cannabinoid receptors in tested mouse groups [53]. In our study, we showed a relationship between the type of cannabinoid, the type of studied cells, and the production of NO. It seems that the release of this molecule is closely related to the type of cells and the current demand for its production.

Cell cycle arrest usually occurs as a result of significant damage to the genetic material of the cell. Repair mechanisms are then activated to remove errors. Sometimes, they cannot be removed, and the cell enters the apoptosis pathway. Cannabinoids are known to inhibit the cell cycle at different stages, depending on the cell type and the type of cannabinoid itself [38]. In our study, we detected significant changes in the number of cells in particular cell cycle phases, depending on the cannabinoid type used. This was confirmed by other authors, who generally found that cannabinoids affected cell cycle pathways via CB1 and CB2 receptors. However, this is not the only way for cannabinoids to limit cancer cell division [17,54]. Afrin et al. also indicated other receptors involved in inhibiting cancer cell cycles, such as TRPV1 [55]. The inhibition of the cell cycle of the cells tested in our study may be thus connected to a different receptor status. Since cannabinoids increase the level of ROS, this simultaneously leads to the occurrence of greater amounts of DNA damage and cell cycle arrest, which we observed in our study. This in turn leads to the induction of apoptosis. It is indicated that the peroxisome proliferator activator receptor c (PPARc) and cannabinoid receptors, especially CB1, play an important role in this process [56].

By controlling cell cycle checkpoints, cannabinoids may strongly influence tumor progression [57]. It has been shown that cell arrest in the G0/G1 phase of the cell cycle may be associated with the activation of ERK1/2 kinases, p27/KIP1 protein induction, and cyclin D1 inhibition [58]. The inhibition in the G1-S transition may be connected with the upregulation of p21 and p27 proteins functioning as CDK inhibitors, the proteolysis of Cdc25A (Cell Division Cycle 25A), being a tyrosine protein phosphatase, and cyclin-dependent kinase 2 (Cdk2) inhibition [59]. Increased expression of the p21 protein functioning as a CDK inhibitor leads to cell cycle arrest in both G1 and G2 phases [60]. Lee H-S et al. showed that CBD application to tumor cells of different origin inhibited the cycle in the G1 phase. Moreover, CBD caused an increase in the sub-G1 cell fraction, which may indicate an increase in the number of cells dying in an apoptotic manner [61]. At the G1-S transition, the cell cycle is inhibited by cannabinoids by limiting AKT activity [62]. The activity of cannabinoids regulating cell cycle checkpoints may therefore be a therapeutic target in cancer treatment [63].

## 5. Conclusions

In summary, cannabinoids exert similar effects on both normal and cancer cells derived from human colon epithelium. The strongest cytotoxic effect was observed for abn-CBG, which is probably due to the presence of a substituent with a conjugated double bond system and the fact that it is structurally the most similar to polyphenols, despite being related to cannabinoids. Depending on the concentration, they had the ability to reduce the free oxygen radical DPPH. Depending on their type, the cannabinoids exerted a varied effect on the production of nitric oxide in both normal and cancer cells of the colon. They influenced the cell cycle, and thus regulated the survival of both normal and cancer cells.

Our study showed the impact of selected cannabinoids on human colon tumor and normal cells. We confirmed the previous information available in the literature regarding the role of these compounds in the regulation of the cell cycle and the induction of cell death, mainly via apoptosis. The novelty of this study was the selection of abn-CBG and CBT, which exhibited high activity in reducing the viability of cancer cells. Unfortunately, however, in the analyzed range of cannabinoid concentrations, normal cells were also damaged and died. Therefore, it seems necessary to determine specific concentrations of cannabinoids that can affect mainly cancer cells, while affecting normal ones to a significantly lesser extent. The advantage of this study is that it indicates that cannabinoids affect human colon epithelial cells, and the adjustment of the concentrations of these substances can bring significant practical effects. Further plans include conducting studies with a longer incubation time and observation of the long-term impact of cannabinoids on human colon cells. In addition, we plan to perform analyses concerning the potential mechanisms of activity of the tested cannabinoids in the presented model. It seems that, due to their interesting properties, the cannabinoids studied may constitute an interesting target for further research, aimed at their use in alternative or combined therapies for human colon cancer.

## Figures and Tables

**Table 1:** IC50 values of the tested cannabinoids after the incubation with colon tumor (HT29) and colon normal cells (CCD 841 CoTr). Results obtained with the MTT method.

Cell Culture	Cannabinoid	IC50 Values (μg/mL)
HT29	CBC	175.96
	CBD	57.54
	abn-CBG	19.61
	CBT	69.1
CCD 841 CoTr	CBC	75.29
	CBD	42.6
	abn-CBG	15.34
	CBT	55.52

**Table 2:** IC50 values of the tested cannabinoids after the incubation with colon tumor (HT29) and colon normal cells (CCD 841 CoTr). Results obtained from the Neutral Red (NR) uptake assay.

Cell Culture	Cannabinoid	IC50 Values (μg/mL)
HT29	CBC	60.57
	CBD	60.5
	abn-CBG	13.66
	CBT	53.89
CCD 841 CoTr	CBC	150.86
	CBD	48.25
	abn-CBG	14.12
	CBT	44.46

**Table 3:** Percentages of viable and dying (apoptosis and necrosis) human colon tumor HT29 cells after 24 h of incubation with the cannabinoids at the 25 μg/mL concentration. A * *p*-value of ≤0.05 is considered significant. The sample results are compared to the untreated control.

Cell Viability Status	Compound				
	Control	CBC	CBD	abn-CBG	CBT
Viable	95.16 ± 0.57	88.03 ± 2.34 *	81.54 ± 0.28 *	4.74 ± 0.39 *	42.91 ± 0.96 *
Early apoptosis	1.10 ± 0.29	1.36 ± 0.18	0.77 ± 0.21	3.65 ± 0.65 *	0.91 ± 0.16
Late apoptosis	3.03 ± 0.71	6.84 ± 0.71 *	17.03 ± 0.29 *	71.86 ± 2.36 *	52.23 ± 1.26 *
Necrosis	0.72 ± 0.28	1.18 ± 0.39	0.67 ± 0.22	19.75 ± 2.50 *	3.96 ± 0.40 *

**Table 4:** Percentages of viable and dying (apoptosis and necrosis) human colon normal CCD 841 CoTr cells after 24 h of incubation with the cannabinoids at the 25 μg/mL concentration. A * *p*-value of ≤0.05 is considered significant. The sample results are compared to the untreated control.

Cell Viability Status	Compound				
	Control	CBC	CBD	abn-CBG	CBT
Viable	92.32 ± 0.52	91.37 ± 1.19	79.37 ± 1.18 *	25.53 ± 1.20 *	87.30 ± 2.11 *
Early apoptosis	1.81 ± 0.21	2.51 ± 0.54	1.97 ± 0.19	8.57 ± 1.67 *	2.90 ± 0.74
Late apoptosis	5.60 ± 0.52	5.58 ± 1.02	16.96 ± 1.26 *	18.36 ± 3.62 *	8.72 ± 1.91 *
Necrosis	0.28 ± 0.04	0.54 ± 0.39	1.71 ± 0.28 *	47.55 ± 4.12 *	1.08 ± 0.56 *

**Table 5:** Percentages of sub-G1, G1, S, and G2 cell cycle phases of HT29 tumor cells after 24 h of incubation with the tested cannabinoids (25 μg/mL). A * *p*-value of ≤0.05 is considered significant. The sample results are compared to the untreated control.

Cell Cycle Phase	Control	Compound			
		CBC	CBD	abn-CBG	CBT
sub-G1	1.33 ± 0.11	2.52 ± 0.10 *	8.59 ± 1.73 *	81.92 ± 1.80 *	19.26 ± 0.67 *
G1	50.04 ± 0.54	50.90 ± 0.73	56.32 ± 2.02 *	7.97 ± 0.35 *	41.86 ± 0.64 *
S	15.61 ± 0.36	16.71 ± 0.37	5.19 ± 0.58 *	5.09 ± 0.50 *	18.64 ± 0.81 *
G2	29.47 ± 0.27	26.23 ± 0.39 *	26.95 ± 2.77 *	3.62 ± 0.54 *	17.90 ± 0.81 *

**Table 6:** Percentages of sub-G1, G1, S, and G2 cell cycle phases of CCD 841 CoTr normal cells after 24 h of incubation with the tested cannabinoids (25 μg/mL). A * *p*-value of ≤0.05 is considered significant. The sample results are compared to the untreated control.

Cell Cycle Phase	Control	Compound			
		CBC	CBD	abn-CBG	CBT
sub-G1	22.13 ± 0.55	15.17 ± 0.79 *	44.01 ± 0.37 *	76.10 ± 2.21 *	28.84 ± 0.66 *
G1	31.92 ± 0.53	32.31 ± 0.62	21.89 ± 0.34 *	11.63 ± 0.83 *	28.81 ± 0.86 *
S	19.66 ± 0.12	23.38 ± 0.79 *	16.39 ± 0.04 *	8.24 ± 0.56 *	22.23 ± 0.15 *
G2	22.29 ± 0.49	22.78 ± 0.86	14.10 ± 0.19 *	3.46 ± 0.79 *	16.54 ± 0.72 *

**Table 7:** Free radical scavenging activity analyzed with the DPPH method. The results are calculated and shown as cannabinoid reduction activity compared to the appropriate Trolox concentration (μg/mL).

Cannabinoid	Cannabinoid Concentration (μg/mL)	Reduction Value Corresponding to the Trolox Concentration (μg/mL)
CBC	25	0
	100	0
	200	0.487 ± 0.341
CBD	25	0
	100	0.471 ± 0.239
	200	0.582 ± 0.363
abn-CBG	25	0.074 ± 0.056
	100	0.216 ± 0.133
	200	0.264 ± 0.198
CBT	25	0.280 ± 0.190
	100	0.487 ± 0.104
	200	0.593 ± 0.278

**Table 8:** Ferric-reducing antioxidant power assay analyzed with the FRAP method. Anhydrous 99.8% methanol was used as a negative control. The results are calculated and shown as cannabinoid reduction activity compared to the appropriate ascorbic acid concentration (μg/mL).

Cannabinoid	Cannabinoid Concentration (μg/mL)	Reduction Value Corresponding to the Ascorbic Acid Concentration (μg/mL)
CBC	25	0.151 ± 0.001
	100	0.453 ± 0.104
	200	1.008 ± 0.403
CBD	25	0.151 ± 0.104
	100	0.202 ± 0.115
	200	0.202 ± 0.163
abn-CBG	25	0.857 ± 0.316
	100	1.059 ± 0.328
	200	1.411 ± 0.465
CBT	25	0.353 ± 0.110
	100	0.353 ± 0.201
	200	0.504 ± 0.258

## Data Availability

The datasets used and/or analyzed during the current study are available from the corresponding author on reasonable request.
